# Sequence Analysis of New *Tuf* Molecular Types of ‘*Candidatus* Phytoplasma Solani’ in Iranian Vineyards

**DOI:** 10.3390/pathogens9060508

**Published:** 2020-06-24

**Authors:** Elham Jamshidi, Sergio Murolo, Mohammad Salehi, Gianfranco Romanazzi

**Affiliations:** 1 Department of Agricultural, Food and Environmental Sciences, Marche Polytechnic University, 60131 Ancona, Italy; e.jamshidi@univpm.it (E.J.); s.murolo@univpm.it (S.M.); 2 Plant Protection Research Department, Fars Agricultural and Natural Resources Research and Education Centre, AREEO, Zarghan 617-71555, Iran; m.salehiabarghuie@areeo.ac.ir

**Keywords:** grapevine, phytoplasma, phytoplasma diseases, stolbur, *tuf*, *Vitis vinifera*

## Abstract

Grapevine Bois noir (BN) is caused by ‘*Candidatus* Phytoplasma solani’ (‘*Ca*. P. solani’) and is one of the most important phytoplasma diseases in the Euro-Mediterranean viticultural areas. The epidemiology of BN can include grapevine as a plant host and is usually transmitted via sap-sucking insects that inhabit herbaceous host plants. Tracking the spread of ‘*Ca.* P. solani’ strains is of great help for the identification of plant reservoirs and insect vectors involved in local BN outbreaks. The molecular epidemiology of ‘*Ca*. P. solani’ is primarily based on sequence analysis of the *tuf* housekeeping gene (which encodes elongation factor Tu). In this study, molecular typing of *tuf*, through restriction fragment length polymorphism and sequencing, was carried out on grapevine samples from Iranian vineyards. According to the molecular characterization, three molecular types—*tuf* b1, *tuf* b5 and *tuf* b6—were found, with *tuf* b1 being the most prominent. These data provide further knowledge of *tuf* gene diversity and question the ecological role of such “minor” *tuf* types in Iranian vineyards, which have been detected only in grapevines.

## 1. Introduction

Grapevine yellow diseases are widespread in many viticultural areas of the world, causing significant economic losses [1], and are very difficult to manage in the field [2]. The most widespread grapevine yellow disease is Bois noir (BN), which is caused by the phytoplasma ‘*Candidatus* Phytoplasma solani’ (‘*Ca*. P. solani’) [3]. This phytoplasma is transmitted from plant to plant mainly by polyphagous planthoppers (e.g., *Hyalesthes obsoletus* and *Reptalus panzeri*) [4,5]. *Urtica dioica* and *Convolvulus arvensis* are the main phytoplasma reservoir plants [6], while grapevine is a dead-end host. 

The molecular epidemiology of ‘*Ca*. P. solani’ is based on the analysis of the house-keeping gene known as *tuf*, which encodes the elongation factor Tu. Molecular characterization of *tuf* by restriction fragment length polymorphism (RFLP) revealed two main types: *tuf* a, which infects stinging nettle (*U. dioca*), and *tuf* b (later called *tuf* b1), which is associated with field bindweed (*C. arvensis*) [7]. A third minor genotype called *tuf* c was detected in hedge bindweed (*Calystegia sepium*) in a restricted area of Germany as an alternative host plant [7,8].

The distribution of the *tuf* types in Europe has also been defined, with *tuf* a being the most common in Germany, Switzerland, Northern France, Northern Italy and Austria, while *tuf* b is spread across most of Europe and Asia. In Iranian vineyards, molecular typing of ‘*Ca*. P. solani’ strains associated with BN led to the identification of the dominant epidemiological cycle of *tuf* b and field bindweed [9].

Using sequence analysis, new *tuf* type variants associated with the nettle and called *tuf* b2 [9,10] or *tuf* ab [11,12], were recently detected in vineyards in Austria, Macedonia, Croatia and Montenegro. Additionally, *tuf* b3 variant was reported in vineyards in the Republic of Azerbaijan [13] and formerly detected in a diseased potato plant collected in Romania [14,15].

The aim of this study was a further characterization of ‘*Ca*. P. solani’ from BN-infected grapevines grown in Iranian vineyards, based on molecular analysis of the *tuf* gene.

## 2. Results

### 2.1. PCR-RFLP and Phylogenetic Analysis

**Figure 1:**
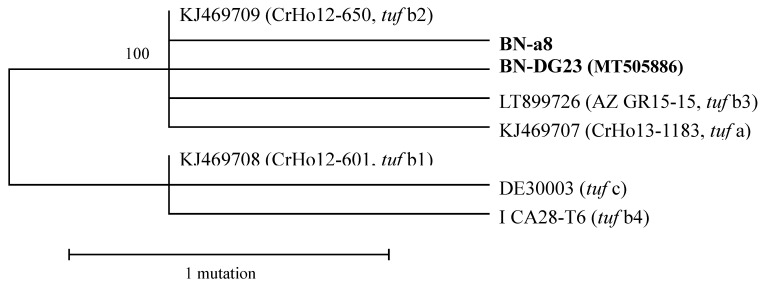
Unrooted phylogenetic tree inferred from the ‘*Candidatus* Phytoplasma solani’ (‘*Ca*. P. solani’) strain nucleotide sequences of the *tuf* gene. Minimum evolution analysis was carried out using the maximum parsimony method. Percentages of replicate trees in which the associated taxa clustered together in the bootstrap test (500 replicates) are shown next to the branches.

According to the 16Sr molecular characterization using a nested PCR and the primer pair fStol/rStol, 85 samples were infected by ‘*Ca.* P. solani’. The positive samples were analyzed with the *tuf* gene primer pairs and further characterized by RFLP with *Hpa*II. Seventy-five samples showed an RFLP profile typical of *tuf* b1, making it the predominant molecular type. Five isolates collected from the Azarbaijan region of Iran and Zanjan province of Iran showed different profiles, distinguishable from the *tuf* a (isolate 19–25) and *tuf* b (isolate P7) reference strains. To support these data, two samples that were representative of the different RFLP patterns were sequenced: BN-a8 and BN-DG23. The BLAST (Basic Local Alignment Search Tool) analysis of the two nucleotide sequences revealed a high sequence homology with ‘*Ca.* P. solani’. The comparison between the *tuf* sequences of the reference strains and the sequences of BN-a8 and BN-DG23 demonstrated two nucleotide substitutions: “A” in position 415 from *tuf* start codon for BN-a8, and “A” in position 444 for BN-DG23, plus a common change for position 727 from “A” to “G”, with respect to the classical *tuf* b1 (Table 1). On the basis of the molecular data, we propose that sequence BN-a8 is referred to as *tuf* b5, and sequence BN-DG23 as *tuf* b6. Phylogenetic analysis indicated significant divergence, due to mutations, among both these new strains and the previous strains (Figure 1).

### 2.2. Mutation Detection

The sequence data were analyzed with the Mutation Surveyor software V5.1.2 (SoftGenetics, State College, PA, USA), using the 2D setting, as this increases the sensitivity and accuracy of the analysis. Figure S1 shows the Mutation Surveyor report that indicated the mutation positions and scores. Two novel mutations were detected in the samples, and some were also found in the reference strains. All of the mutations were substitution mutations. The most common mutations were detected for position 727. According to this position, there are two groups of *tuf* types: *tuf* a, *tuf* b2, *tuf* b3, *tuf* b5, and *tuf* b6 with a substitution from “A” to “G”, and *tuf* b1, *tuf* b4, and *tuf* c with a substitution from “G” to “A”.

## 3. Discussion

Prokaryotic and eukaryotic elongation factors are essential in the formation of peptide bonds during protein synthesis [16]. The bacterial elongation factor Tu is a GTP-binding protein that has a central role in protein synthesis, is highly conserved throughout bacteria and is homologous to its eukaryotic counterpart. The *tuf* gene is a single-copy gene in phytoplasmas that displays more variations than the 16S rRNA gene and is therefore a good candidate for phytoplasma classification [17]. This conserved gene encodes the elongation factor Tu that has a central function in translation processes [18].

Initially, characterization of ‘*Ca*. P. solani’ according to RFLP analysis of *tuf* identified three genetic types as *tuf* a, *tuf* b, and *tuf* c [7]. *U. dioica* is considered the dominant reservoir plant for *tuf* a*,* and *C. sepium* for *tuf* c [6]. However, for *tuf* b, while field bindweed is the dominant reservoir plant, several other herbaceous plants that can be found in and around infected vineyards have been reported as being hosts for ‘*Ca.* P. solani’, including *Chenopodium album*, *Tussilago farfara* and *Malva sylvestris*; therefore, these might all have key roles in the spread of BN [19,20,21,22,23]. The *tuf* b strain of ‘*Ca*. P. solani’ has also been identified in an epidemiological setting involving *Vitex agnus-castus*, with transmission to grapevine via associated populations of *H. obsoletus* [12,24]. Recent studies have also reported on a further independent epidemiological cycle for the *tuf* b strain of ‘*Ca*. P. solani’, detected in *Crepis foetida* and with transmission by *H. obsoletus* [25]. However, this study detected *tuf* b1 and two new genotypes that have not been reported previously that differ from *tuf* b1 in mutations. For BN-a8, the substitution in position 415 after the ATG start codon results in a more stable amino acid leading the synthesis of isoleucine instead of leucine. The substitution at position 444 for the BN-DG23 strain does not result in a change in amino acid. These new two *tuf* types were recorded in grapevine samples, and we can presumably hypothesize that they are present in wild plants and insects, which occasionally feed on grapevine. It is well known that diverse functions have been ascribed to the elongation factor Tu, many of which include important functions in the bacterial cytosol and for the cell surface [26,27,28,29]. Functions such as adhesion to host cells and molecules are fundamental to pathogenesis in many bacterial species as it facilitates colonization, invasion and host immune suppression [30]. Therefore, the different types of *tuf* gene generated by nucleotide modification might lead to a variation in amino acid level, hence playing a key role as fitness factors by extending or reducing the range of hosts. 

These findings support the hypothesis that the *tuf* gene is involved in interactions of ‘*Ca*. P. solani’-related strains with their host plants and/or their insect vectors, which can drive the adaptation of diverse phytoplasma genetic lineages to varied vineyard ecosystems. In conclusion, in Iranian vineyards, the main *tuf* type is *tuf* b1, but we describe here the new *tuf* types of *tuf* b5 and *tuf* b6 that have emerged. Future research about their ecological and epidemiological roles in vineyard ecosystems should be conducted using innovative tools based on geostatistical analysis [31].

## 4. Materials and Methods

### 4.1. Plant Samples and DNA Extraction

A total of 124 grapevine samples were collected during surveys carried out from mid-September to the beginning of October in 2016 and 2017 in local vineyards which constituted of white and red varieties. This intensified the phytosanitary monitoring for grapevine yellows carried out in 2015 in vineyards located in the Azarbaijan Gharbi, Azarbaijan Sharghi, Zanjan, Qazvin, Fars, Lorestan and Khorasan Razavi provinces of Iran, as previously reported by Jamshidi et al. [9]. Total nucleic acids were extracted from 1 g fresh petioles of symptomatic grapevines from old and young leaves using the cetyltrimethylammonium bromide method, as described by Daire et al. [32]. The concentrations and purities of the DNA samples were determined using a spectrophotometer (Biophotometer Plus, Eppendorf, Hamburg, Germany). 

### 4.2. PCR Amplification, RFLP and Sequencing

The PCR was performed in a programmable Bio-Rad Cycler (Bio-Rad, Berkeley, CA, USA), using the universal P1/P7 phytoplasma primer pair, followed by a nested PCR with the rStol/fStol specific primer pair for stolbur phytoplasma [33]. DNA amplification was performed in 20 μL volumes and PCR products were separated on 1% agarose gel, stained with GelRed (Biotium Inc., Fremont, CA, USA) and visualized with a UV transilluminator. The *tuf* gene was amplified using Tuf1f/r, followed by the TufAYf/r primer in a nested PCR [6,7]. Nested PCR amplicons (940 bp) were subjected to restriction digestion using *Hpa*II endonuclease (ThermoScientific, Darmstadt, Germany), following the manufacturer’s instructions. The restriction products were separated by electrophoresis in 2.5% agarose gel. Samples, amplified with tufAYf/r primer pairs and showing a different restriction pattern compared to *tuf* a and *tuf* b reference isolates (19–25 and P7) were selected and sent for sequence analysis at the Genewiz Genomics sequencing service (Genewiz UK, Takeley, UK: https://www.genewiz.com/).

### 4.3. Phylogenetic Analysis

The nucleotide sequences of BN-a8 and BN-DG23 were subjected to BLAST analysis to confirm their identities. Multiple alignments of the nucleotide and amino acid sequences were carried out using ClustalX [34]. The phylogenetic relationships were calculated using the maximum parsimony method. The percentages of replicate trees in which the associated taxa clustered together in the bootstrap test, with 500 replicates, are shown next to the branches. The maximum parsimony tree was obtained using the subtree pruning and regrafting algorithm. A total of 901 positions were found in the final dataset. Evolutionary analyses were conducted using the MEGA (Molecular Evolutionary Genetics Analysis) 6 software (http://www.megasoftware.net/index.html).

### 4.4. Data Analysis

The sequence data obtained were loaded into Mutation Surveyor V5.0.1 (SoftGenetics, State College, PA, USA), a software specifically designed to detect low-frequency DNA variants from sequenced data. The mutation score was used by the software to recognize a mutation and to rank its confidence level, a measure of the probability of error, which was based on the ratios of the noise level, the overlapping factor and the dropping factor, as calculated by the software. Mutations were investigated in nucleotide reference strains and in the samples found in this study. The sequences of BN-a8 and BN-DG23 were analyzed and compared with reference sequences that were already available in the NCBI database (https://www.ncbi.nlm.nih.gov/), including: *tuf* a (CrHo13-1183), *tuf* b1 (CrHo12-601), *tuf* b2 (CrHo12-650) [8], *tuf* b3 (AZ_GR15-156 15) [12], *tuf* b4 (I CA28-T6) (Murolo and Romanazzi, unpublished) and *tuf* c (DE 30003) (Langer and Maixner, unpublished). The Mutation Surveyor software was set for 2D (bi-directional) small peaks; the mutation-calling parameters were set to the defaults, including for the overlapping factor and the dropping factor. The overlapping factor was calculated by the software from the two different bases in the reference and sample traces on either side of the mutation. The dropping factor was determined from the relative intensities of the four neighboring peaks (i.e., two peaks on each side) between the sample traces and the reference traces.

## Figures and Tables

**Table 1:** Mutations identified for the different *tuf* types, with respect to *tuf* b1 (Mutation Surveyor (MS) analysis).

Accession Number	Nucleotide Sequence ID	*Tuf* Type	Nucleotide Substitution (MS Report)	Position from *Tuf* Start Codon	Codon Change	Amino-Acid Change	Mutation Type	Strain Reference
MT505885	BN-a8	*tuf* b5	C>A	415	CTA/ATA	Lue/Ilue	NS ^a^	Current study
			A>G	727	AAA/AAG	Lys/Lys	S ^b^	
MT505886	BN-DG23	*tuf* b6	G>A	444	GTG/GTA	Val/Val	S	Current study
			A>G	727	AAA/AAG	Lys/Lys	S	
KJ469707	CrHo13-1183	*tuf* a	T>C	666	CCG/CTG	Lue/Pro	NS	[6]
			A>G	727	AAA/AAG	Lys/Lys	S	
KJ469708	CrHo12-601	*tuf* b1	G>A	727	AAG/AAA	Lys/Lys	S	[6]
KJ469709	CrHo12-650	*tuf* b2	A>G	727	AAA/AAG	Lys/Lys	S	[8]
LT899726	AZ_GR15-15	*tuf* b3	A>G	727	AAA/AAG	Lys/Lys	S	
			T>C	917	TGG/CGG	Trp/Arg	NS	[13]
Unpublished	I CA28-T6	*tuf* b4	C>T	570	ACG/ATG	Thr/Met	NS	
			G>A	727	AAG/AAA	Lys/Lys	S	Unpublished
Unpublished	DE30003	*tuf* c	A>G	642	CAG/CGG	Gly/Arg	NS	
			G>A	727	AAG/AAA	Lys/Lys	S	Unpublished

^a^ nonsynonymous mutation. ^b^ synonymous mutation.

## Supplementary Materials

The following are available online at https://www.mdpi.com/2076-0817/9/6/508/s1, Figure S1: Sequence analysis results using Mutation Surveyor V5.1.2, Graphic Analysis display for single nucleotide changes in the a8 and DG23 samples, compared with the reference strains.
