# Supplementary material for: Sequence Analysis of New Tuf Molecular Types of ‘Candidatus Phytoplasma Solani’ in Iranian Vineyards
Source: Pathogens. 2020 Jun 24;9(6):508. doi: 10.3390/pathogens9060508 (PMC7350298; doi:10.3390/pathogens9060508)
Supplement: Supplementary file 1 [file pathogens-09-00508-s001.pdf]

Article

Sequence Analysis of New *Tuf* Molecular Types of ‘*Candidatus* Phytoplasma Solani’ in Iranian Vineyards

Elham Jamshidi [1], Sergio Murolo [1], Mohammad Salehi [2] and Gianfranco Romanazzi [1,*]

1 Department of Agricultural, Food and Environmental Sciences, Marche Polytechnic University, 60131 Ancona, Italy; e.jamshidi@univpm.it (E.J.); s.murolo@univpm.it (S.M.)

2 Plant Protection Research Department, Fars Agricultural and Natural Resources Research and Education Centre, AREEO, Zarghan 617-71555, Iran; m.salehiabarghuie@areeo.ac.ir

* Correspondence: g.romanazzi@univpm.it; Tel.: +39 071 2204336

Received: 22 April 2020; Accepted: 17 June 2020; Published: date

Supplemental material

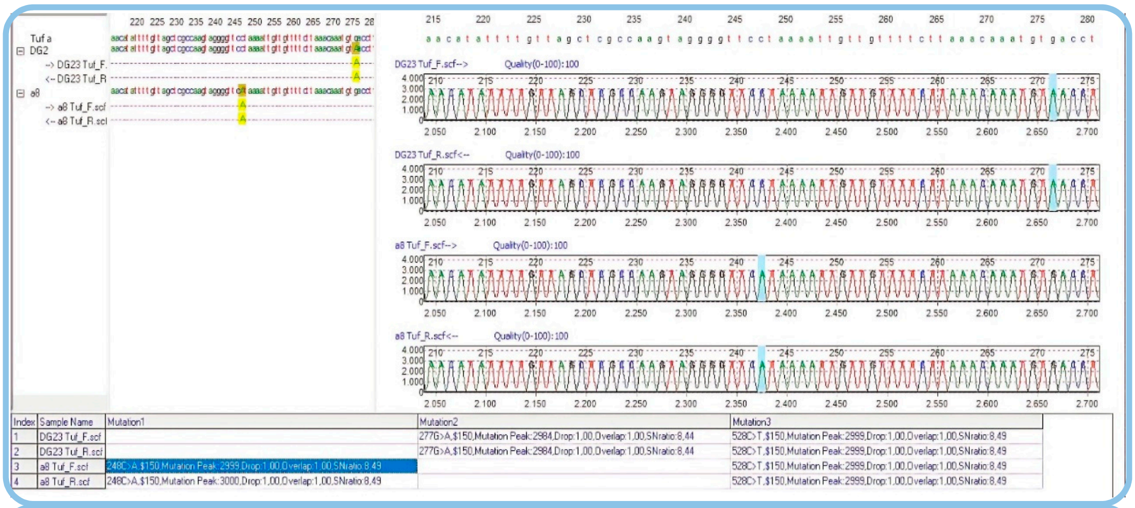

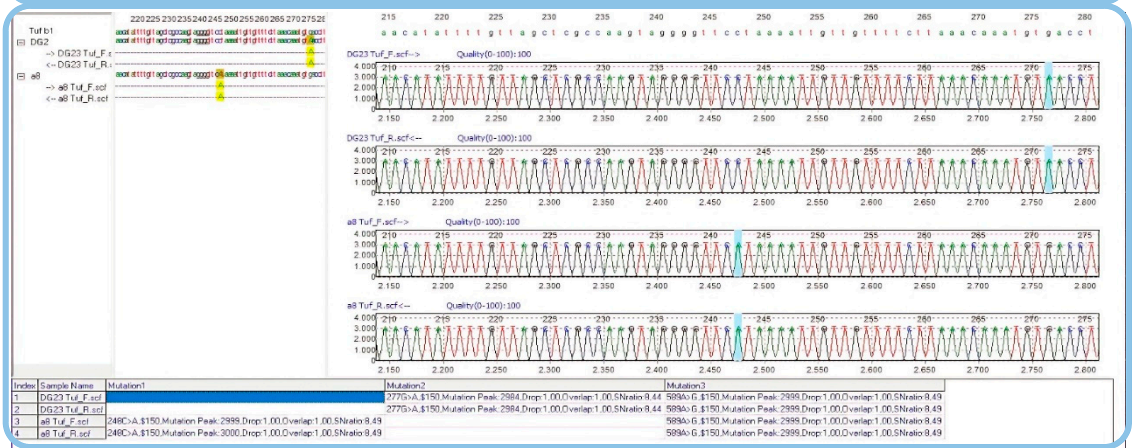

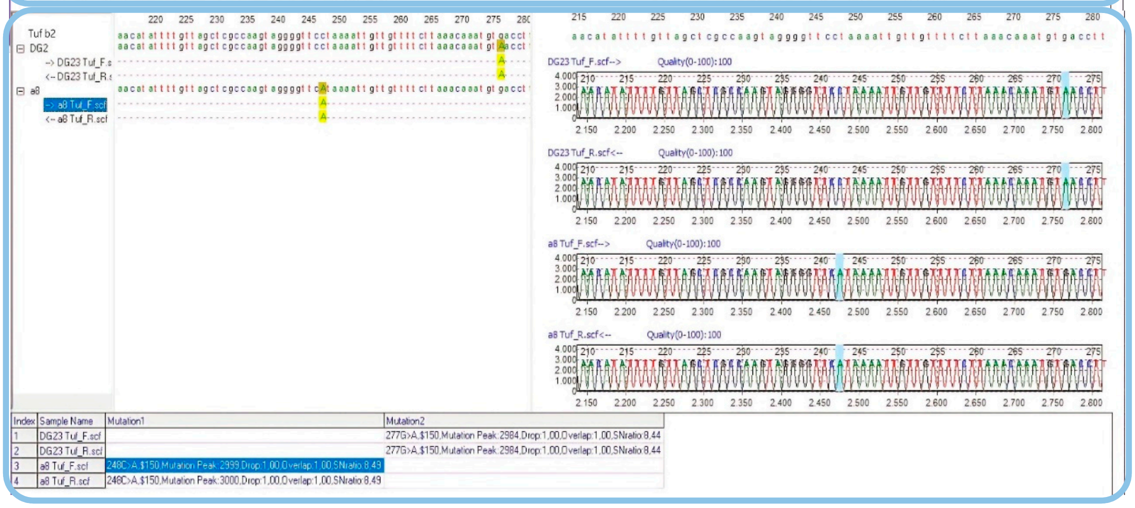

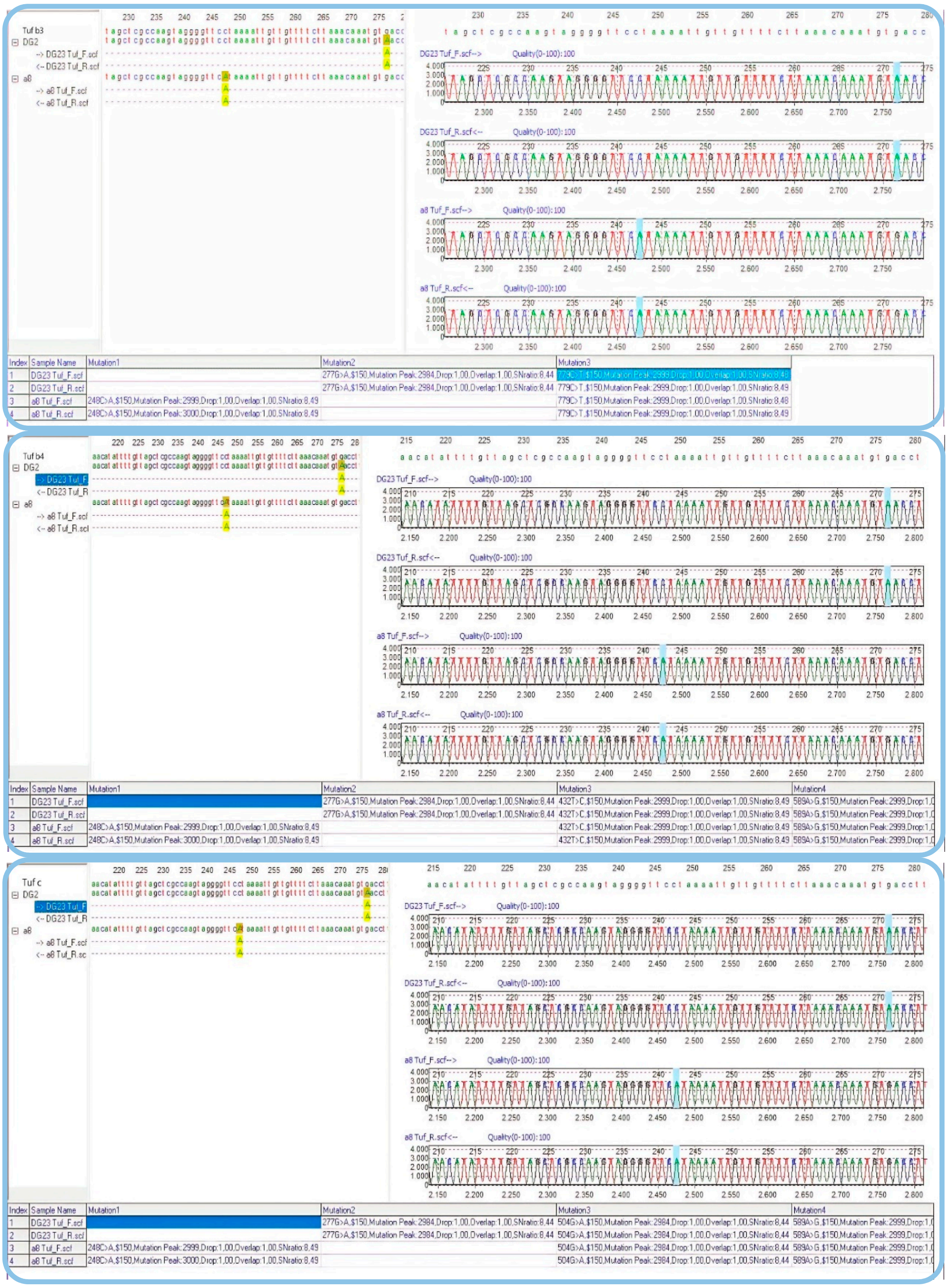

Figure 1. Sequence analysis results using Mutation Surveyor V4.0.8, Graphic Analysis display for single nucleotide changes in the a8 and DG23 samples, compared with the reference strains.
